# The Lodwick classification for grading growth rate of lytic bone tumors: a decision tree approach

**DOI:** 10.1007/s00256-021-03868-8

**Published:** 2021-07-24

**Authors:** Matthias Benndorf, Fabian Bamberg, Pia M. Jungmann

**Affiliations:** Department of Radiology, Medical Center - University of Freiburg, Faculty of Medicine, University of Freiburg, Hugstetter Straße 55, 79106 Freiburg, Germany

**Keywords:** Bone tumor, Growth rate, Lodwick, Decision tree

## Abstract

The estimation of growth rate of lytic bone tumors based on conventional radiography has been extensively studied. While benign tumors exhibit slow growth, malignant tumors are more likely to show fast growth. The most frequently used algorithm for grading of growth rate on conventional radiography was published by Gwilym Lodwick. Based on the evaluation of the four descriptors (1) type of bone destruction (including the subdescriptor “margin” for geographic lesions), (2) penetration of cortex, (3) presence of a sclerotic rim, and (4) expanded shell, an overall growth grade (IA, IB, IC, II, III) can be assigned, with higher grade representing faster tumor growth. In this article, we provide an easy-to-use decision tree of Lodwick’s original grading algorithm, suitable for teaching of students and residents. Subtleties of the grading algorithm and potential pitfalls in clinical practice are explained and illustrated. Exemplary conventional radiographs provided for each descriptor in the decision tree may be used as a guide and atlas for assisting in evaluation of individual features in daily clinical practice.

**Supplementary Information:**

The online version contains supplementary material available at 10.1007/s00256-021-03868-8.

## Introduction

The evaluation of bone tumors with plain radiography has been extensively studied. The inherently strong contrast between bone and soft tissue and the high spatial resolution of radiography allow for the evaluation of even subtle morphological distortions of bone structure [1]. In the context of evaluating a bone tumor with radiography, the radiologist’s task is twofold: to provide information on (1) growth rate and (2) entity of the lesion, respectively. The first task of providing information on the growth rate is of major importance [2, p. 59], since fast growth rates indicate malignancy of the bone tumor [3]. Fast growth rates estimated with radiography are known to be associated with decreased patient survival and poorer prognosis [4]. Besides assessing a tumor’s growth rate longitudinally on serial images, it is also possible to estimate growth rate from the radiographic appearance at a single time point [4], since the reaction pattern of the local bone tissue depends on the growth rate of the tumor. Therefore, radiography remains an indispensable modality for the evaluation of bone tumors.

Due to the low incidence of bone tumors, the individual general radiologist cannot hope to acquire sufficient expertise in estimating growth rates on conventional radiography and in providing accurate diagnoses simply based on personal experience [2, p. 3,5]. Therefore, classification systems have been developed in order to help radiologists completing these tasks. The classification system developed in 1980 by Gwilym Lodwick [4] has become the textbook standard for grading bone tumor growth [6]. In daily clinical practice, the applied classification is frequently referred to as such (“Lodwick grades I–III”). However, it has been argued that the original Lodwick classification system is complex and requires simplification [3].

Therefore, the aims of the present article are (1) to provide an easy-to-use decision tree of the original Lodwick grading algorithm suitable for teaching of students and residents with an illustration that may be used in clinical practice, (2) to provide exemplary conventional radiographs for each descriptor in the decision tree as a guide and atlas for assisting in grading and evaluation of individual features, and (3) to point out subtleties and potential pitfalls with imaging examples in order to improve the correct application.

## The decision tree

**Fig. 1:**
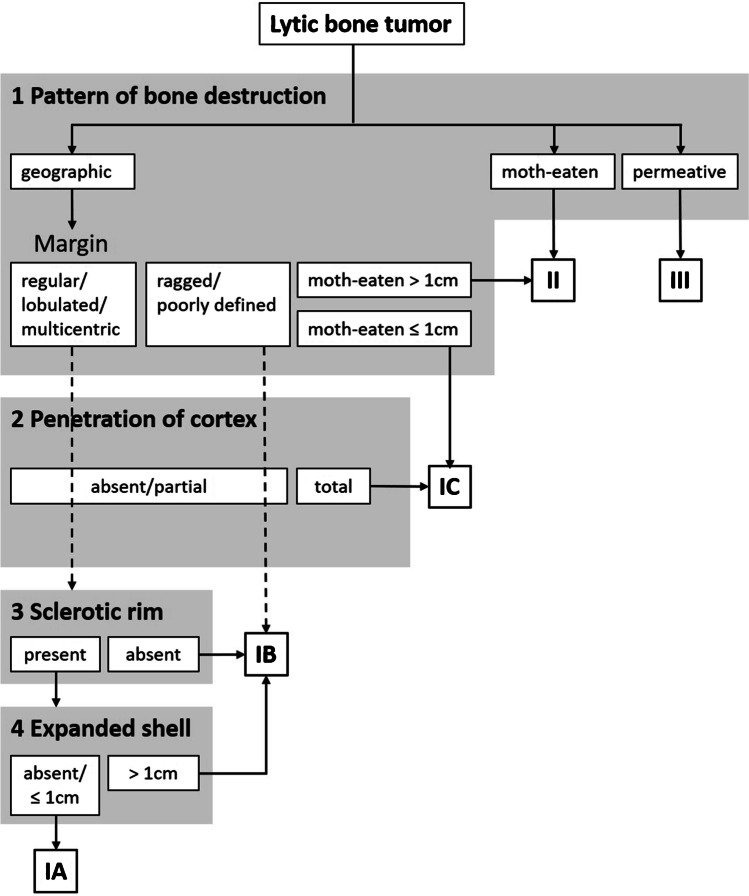
Decision tree to derive the Lodwick growth grade of lytic bone tumors, following the original truth table from [4]. The intended sequence of descriptor evaluation has been preserved (gray boxes). Penetration of cortex is evaluated for geographic lesions without moth-eaten margin. An absent/partial penetration leads either to further evaluation of presence of a sclerotic rim or directly to growth grade IB, depending on the respective margin. Total penetration of cortex prompts assignment of growth grade IC

In the Lodwick grading algorithm, four descriptors were selected for evaluation of a lytic bone tumor on radiography [4]. These comprise (1) pattern of bone destruction, including the subdescriptor “margin” for the category geographic destruction; (2) penetration of cortex; (3) sclerotic rim; and (4) expanded shell. The descriptors with their subcategories and definitions/explanations are provided in Table 1 (with reference to Table 8 from [4]). These four descriptors are the basis for the following sequential categorization, finally resulting in the growth grades IA, IB, IC, II, and III [2, 4]. The higher the category, the faster the tumor is considered to grow. Instead of tables, as provided in the original publication by Lodwick and colleagues, a decision tree is shown in Fig. 1 that follows the originally intended sequential evaluation of these four descriptors in a specific order. This decision tree includes the entire original information of the grading algorithm [4], but is designed for application in clinical practice. Regarding the pattern of bone destruction (descriptor 1), a geographic pattern is defined as “single or confluent hole in bone,” a moth-eaten pattern is defined as “multiple, apparently randomly distributed holes which […] lack uniformity of size,” and a permeative pattern is defined as “multiple uniformly small holes present anywhere in the lesion” [4]. For the geographic pattern, additional evaluation is required regarding the margin of the lesion.

**Table 1:** Descriptors employed by the Lodwick grading algorithm for bone tumor growth rate, following Table 8 from [4]

Descriptor	Commentary
Pattern of bone destruction	
Geographic	Definition geographic^1^:
With regular margin	“Single or confluent hole in bone”
With lobulated margin	
With multicentric margin	
With ragged or poorly defined margin	
With moth-eaten margin ≤ 1 cm	
With moth-eaten margin > 1 cm	
Moth-eaten	Definition moth-eaten^1^:
	“Multiple, apparently randomly distributed holes which […] lack uniformity of size”
Permeative	Definition permeative^1^:
	“Multiple uniformly small holes” automatically evaluated as permeative when present anywhere in the lesion
Penetration of cortex	
Absent	For growth grades II und IIII, total penetration of cortex is assumed
Partial	
Total	
Sclerotic rim	
Present	
Absent	
Expanded shell	
Absent	
≤ 1 cm	
> 1 cm	

^1^The definitions of bone destruction patterns are reproduced literally in an abbreviated manner from [4]

The merit of our representation of the grading algorithm is the intuitive exclusion of growth grades that are not compatible with observed descriptors. In the original truth table and plain text table, information for all descriptors and all growth grades is provided—although at specific points in the sequence of evaluation, growth grades are eliminated from further consideration [4].

## Atlas of descriptors

**Fig. 2:**
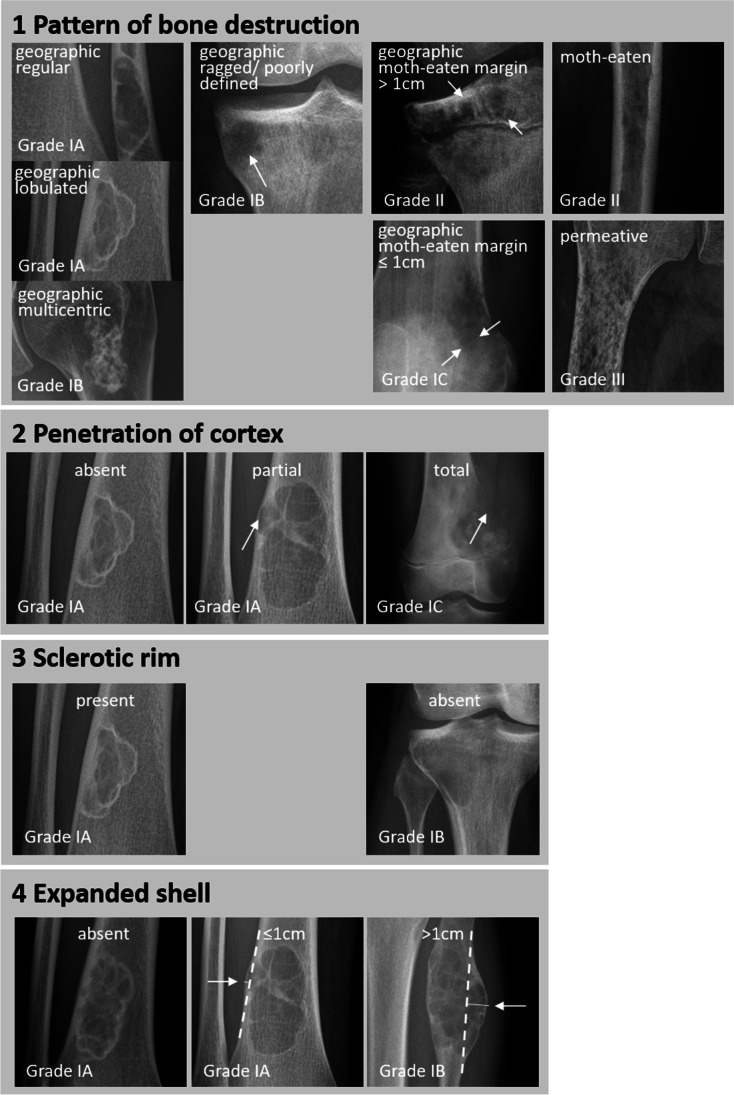
Radiographic examples of the descriptors employed by the Lodwick grading algorithm for bone tumor growth rate

Exemplary conventional radiographs that show all descriptors used by the Lodwick grading algorithm are provided in Fig. 2. In addition, the finally assigned Lodwick growth grade of the respective lesions is provided in the images.

## Pitfalls

**Fig. 3:**
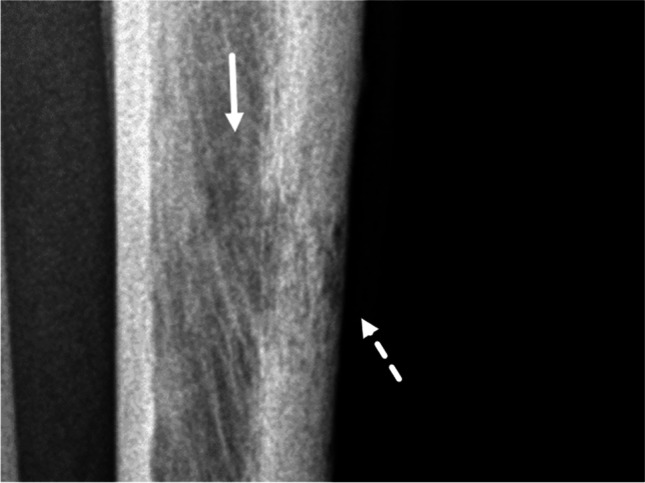
Grade III. A 74-year-old man. A barely visible osteolytic bone tumor is observed in the diaphysis of the left tibia (solid arrow); margins are poorly defined. There is permeative tumor growth in the adjacent lateral cortex (dashed arrow). This latter point prompts the assignment of Lodwick growth grade III, irrespective of any descriptor of the adjacent lucency. The tumor proved to be a metastasis from a caecal carcinoma

**Fig. 4:**
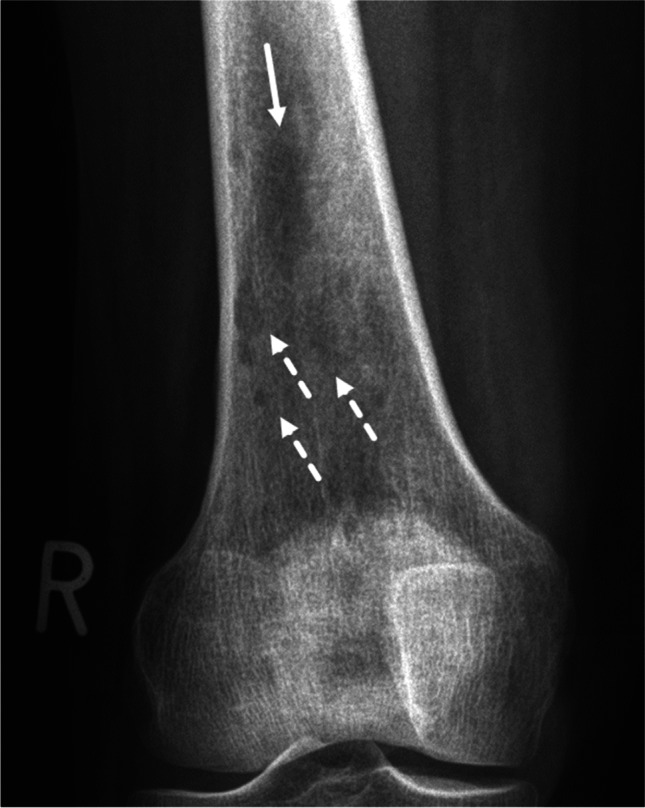
Grade II. A 54-year-old woman. One larger ill-defined lytic bone tumor is observed (solid arrow). Distal to this lesion are several round/oval smaller lytic lesions of varying size (dashed arrows). This is a moth-eaten pattern of bone destruction, i.e., Lodwick growth grade II is assigned. Note that if the larger lesion is considered the dominant (geographic) lesion, the overall resulting growth grade is still II—due to the width of the moth-eaten margin distally. This patient had multiple myeloma

**Fig. 5:**
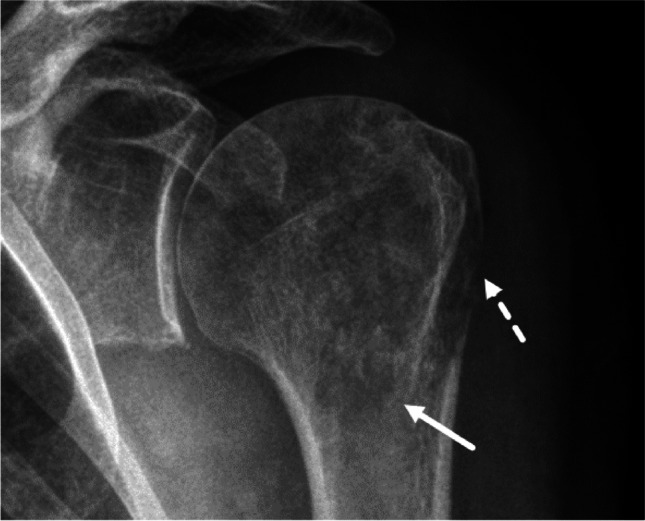
Grade IC. A 71-year-old woman. A geographic lytic bone tumor in the proximal left humerus is observed. The tumor is geographic in nature and has a ragged and poorly defined margin (solid arrow). Total penetration of cortex is evident (dashed arrow) and results in assignment of Lodwick growth grade IC. The evaluation regarding a sclerotic rim and an expanded shell is not necessary to assign this growth grade. The tumor proved to be diffuse large B-cell non-Hodgkin Lymphoma

**Fig. 6:**
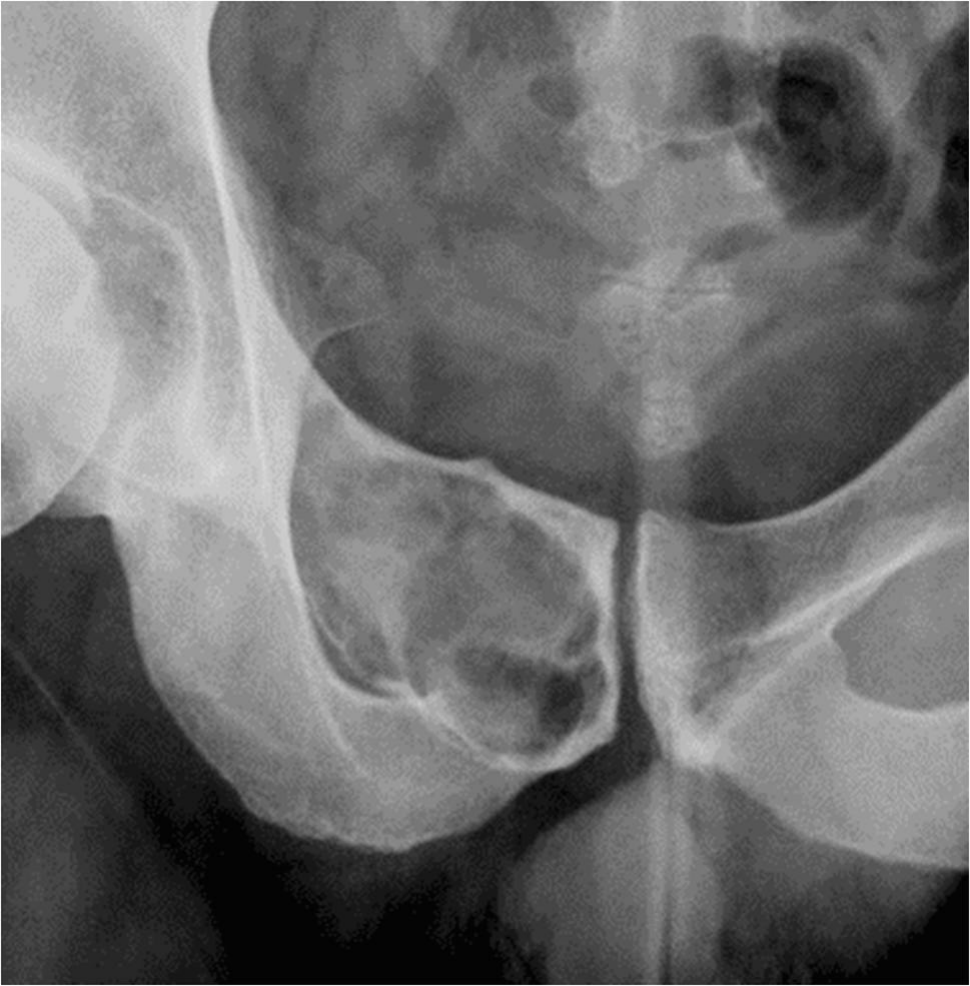
Grade IB. A 32-year-old man. A well-defined lytic bone tumor in the right superior pubic ramus is observed. The tumor is geographic in nature and has a regular to lobulated margin. There is thinning of the expanded shell, but no total cortical penetration. The shell is expanded beyond 1 cm of what is considered the normal contour of the superior pubic ramus (compare for contralateral side). Therefore, Lodwick growth grade IB is assigned. The tumor proved to be a chondromyxoid fibroma

**Fig. 7:**
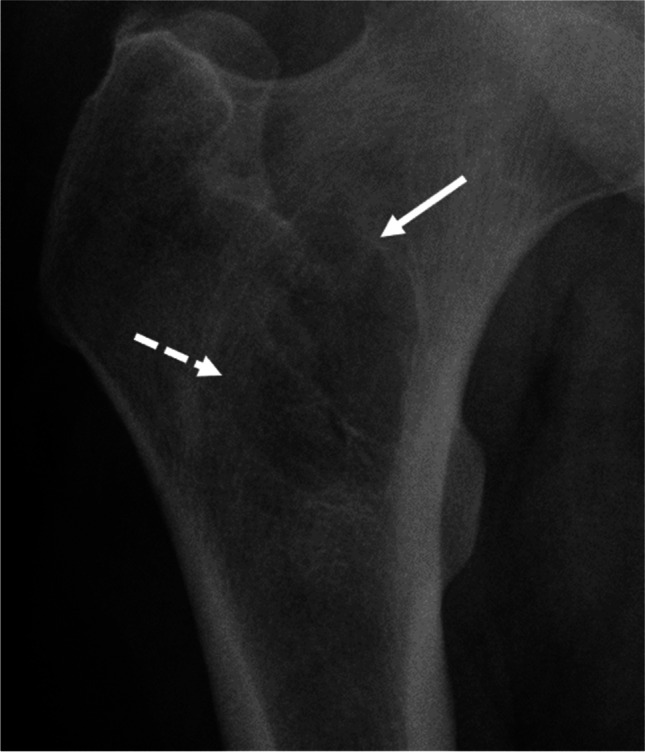
Grade IB. A 43-year-old man. A well-defined tumor is observed in the proximal femur. No penetration of cortex is evident. A faint sclerotic margin is observed in parts of the lesion (solid arrow); other parts of the lesion do not show a sclerotic margin (dashed arrow). There is no expanded shell. Because the sclerotic margin is not visible around the entire lesion, Lodwick growth grade IB is assigned. MRI was performed for further evaluation; the lesion proved to be a lipoma

**Fig. 8:**
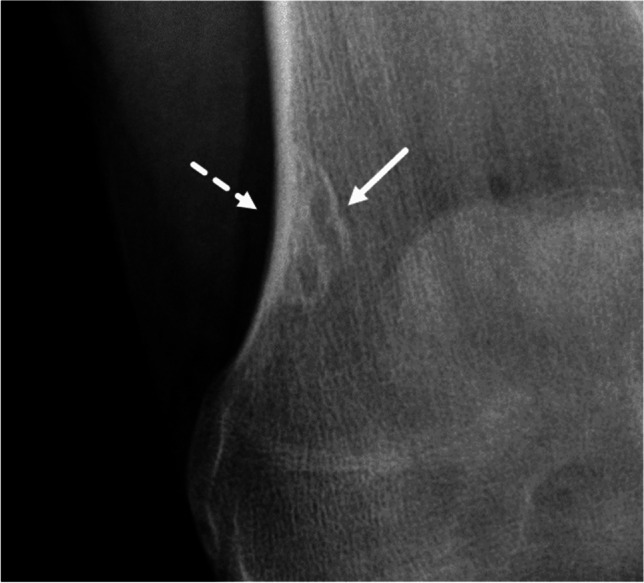
Grade IA. A 20-year-old woman. A lobulated, sharply marginated geographic tumor is observed in the medial distal metaphysis of the femur. There is no penetration of cortex; a sclerotic rim is observed (solid arrow). The contour of the cortex is normal (no expanded shell, dashed arrow). This is the combination of descriptors that leads to assignment of Lodwick growth grade IA. The appearance is considered typical for non-ossifying fibroma (NOF); a histological diagnosis is not warranted in such a case [25]

With reference to the original publication, there are several aspects in the diagnostic algorithm that require special consideration and potentially represent pitfalls (Table 2). These refer to geographic lesions. Examples of the application of the decision tree with reference to the respective pitfalls are presented in Figs. 3, 4, 5, 6, 7, and 8. For each case, the radiograph, the observed descriptors, and the reasoning that leads to final growth grade assignment are presented.

1. It is possible that a mostly geographic lesion is classified as growth grade III. To assign growth grade III, the permeative pattern only needs to be present in part of the lesion [2 pp. 61–3] (Fig.3).
2. It is possible that a geographic lesion is classified as growth grade II. This happens if a moth-eaten margin > 1 cm is observed (Fig.4).
3. If there is total penetration of cortex in a geographic lesion, growth grade IC is assigned, irrespective of the actual margin descriptor (regular/lobular/multicentric/ragged/poorly defined). A thin moth-eaten margin also leads to assignment of growth grade IC (Fig.5).
4. If there is an expanded shell > 1 cm in a geographic lesion without a moth-eaten margin, without/partial penetration of cortex, and with a sclerotic rim, growth grade IB is assigned (Fig.6). The extent of the expanded shell is the only difference to growth grade IA lesions in these cases (see Pitfall 6., Fig. 8).
5. Growth grade IB is also assigned if there is a geographic lesion with an incomplete sclerotic rim, without a moth-eaten margin and without/partial penetration of cortex (Fig.7). Here we follow the reasoning of Caracciolo and colleagues that “it is a fundamental principle of lesion analysis that margins are classified by their most aggressive features” [3].
6. There is only one distinct combination of descriptors that allows for the assignment of growth grade IA: regular/lobulated/multicentric margin, no/partial penetration of cortex, sclerotic rim, no or slightly expanded (≤ 1 cm) shell (Fig.8).

**Table 2:** Potential pitfalls of the Lodwick grading system

Image impression	Lodwick growth grade	Explanation	Figure
Geographic lesion with small permeative part	Grade III	As soon as a permeative part is observed in a lytic bone lesion, the lesion is considered grade III, even if most of the lesion is geographic	3
Geographic lesion with a moth-eaten margin > 1 cm	Grade II	Not only an entirely moth-eaten lesion, but also a geographic lesion with a moth-eaten margin > 1 cm is considered as grade II. If a moth-eaten margin ≤ 1 cm is observed in a geographic lesion it is considered grade IC	4
Geographic lesion with total penetration of cortex	Grade IC	As soon as there is total penetration of cortex any geographic lesion except those with a moth-eaten margin > 1 cm (grade II) are considered grade IC	5
Geographic lesion without/partial penetration of cortex and expanded shell > 1 cm	Grade IB	Partial penetration of cortex is allowed for assignment of grade IA or IB. An expanded shell > 1 cm leads to assignment of grade IB	6
Geographic lesion with an incomplete sclerotic rim	Grade IB	A geographic lesion with an incomplete sclerotic rim and without/partial penetration of cortex is considered grade IB	7
Geographic lesion with regular/lobulated/multicentric margin, no/partial penetration of cortex a sclerotic rim and no or slightly expanded (≤ 1 cm) shell	Grade IA	This is the only lesion type that is assigned grade IA	8

## Structured reporting

To further improve consistency of reporting of lytic bone tumors, we provide predefined expressions as supplemental material (supplement 1) that may be used in written radiology reports. At this stage, we only include information that is required by the Lodwick algorithm to derive the corresponding growth grade. Additional, important information for deriving differential diagnoses (like tumor localization in the bone, the presence of matrix calcifications, or type of periosteal reaction) should still be described in the radiological report and may be part of future investigations.

## Discussion

### Summary

The Lodwick grading system is the most frequently applied algorithm for evaluation of the growth rate of lytic bone tumors and consequently for estimation of probability of malignancy and prognosis. In the present work, an easily comprehensible decision tree following the diagnostic pathway defined by the original Lodwick grading algorithm is provided. Corresponding exemplary radiographs of all employed descriptors are provided that may be used as an atlas. Furthermore, subtle details of the grading algorithm that are little-known and may result in misclassification of lesions are highlighted. Therefore, the figures and tables are potentially useful for students, residents, and radiologists in clinical practice, hopefully resulting in reduced misclassification and better interobserver agreement.

### History of Lodwick’s work

The evaluation of bone tumors with radiography was substantially improved by Gwilym Lodwick. He introduced an algorithm that allows accurate grading of growth rate of bone tumors feasible in everyday practice of musculoskeletal radiologists. The grading algorithm has undergone refinement by Gwilym Lodwick himself during his career. In the first publication from 1964, the initially suggested grading system was presented in text table format and also included the parameters *buttress*, *Codman’s triangle*, and *tumor bone* [7]. In 1971, in the most extensive iteration, also, non-lytic lesions were graded—by relying on the presence of *reactive bone phenomena*, *tumor matrix calcifications*, and *tumor size* [2, p. 63]. Also, the parameters *mottling* (defined as thickened trabeculae) and *ground glass opacity* were included [2, p. 60]. Radiology textbooks and the present manuscript refer to his latest classification system from 1980 that focused on fewer descriptors and on tumors with evident bone destruction [4].

### Algorithm format

The original Lodwick grading algorithm from 1980 was presented in truth table format [4]. In the truth table, beside the list of descriptors, the information is provided whether presence of the descriptor is compatible with growth grades IA, IB, IC, II, and III (true or false, respectively; Boolean). In addition, the information was also provided as plain text table [4], which is reproduced in radiology textbooks e.g., [6, 8, 9]. In comparison, irrelevant information was eliminated in the presented decision tree. Whenever a growth grade is assigned “false” in the truth table, this grade is eliminated from further consideration in the diagnostic process. Owing to the design of the truth table, information for excluded growth grades is still mentioned further down in the table [4]. The decision tree in the present work is designed such that there is no necessity to keep track of already excluded growth grades that do not require further consideration. Although Lodwick considered the original format applicable in clinical routine, truth table and plain text table formats are not appealing for clinical application. The simplification and the visually appealing format of the decision tree and the exemplary images in the present manuscript may improve clinical applicability of the Lodwick grading system and reduce misclassification.

### Subsequent works

Since the original publication, two adjusted variants of the Lodwick grading algorithm have been proposed. In 1981, Madewell introduced the descriptor of a *changing margin* [10] in a longitudinal observation. Madewell additionally introduced *combination patterns*, where features of different growth grades are present at a single time point [10]. In 2016, Caracciolo and colleagues published a “Modified Lodwick-Madewell Grading System,” further refining the parameter *changing margin* [3]. They suggested to introduce a new category IIIA for lesions with changes in margins on serial radiographs or for atypical combinations suggestive of malignancy when prior radiographs are not available, respectively [3]. This category IIIA implies a high probability of malignancy. Furthermore, grade IC becomes grade II to account for the moderate risk of malignancy of these lesions. Grade IIIB of this system contains Lodwick grades II and III. Radiographically occult lesions are assigned category IIIC [11]. Neither modified system has been demonstrated to be superior to the original Lodwick grading system of 1980.

### Reproducibility and accuracy of diagnosis

Regarding reproducibility, it was demonstrated that readers were more frequently agreeing in assignment of growth grades IA (most likely to be benign) and III (most likely to be malignant) as compared to the assignment of intermediate grades [12]. Considerable accuracy was reported for correlations of growth rates with malignancy: 94% of grade I lesions were benign and 81% of grade III lesions were malignant when evaluated with the adjusted variant of the Lodwick grading algorithm by Caracciolo and colleagues [3]. In addition, most referring clinicians expect the radiologist to provide differential diagnoses, ranked by probability. The overall accuracy of predicting the correct histopathological tumor type based on radiographic imaging features and demographical data ranges between 44 and 78% [5, 13, 14]. The referenced studies employed a naïve Bayes classifier to rank differential diagnoses by probability. Naïve Bayes classifiers derive posttest probabilities given the values of several predictive variables, all of which are regarded conditionally independent. For a detailed methodological discussion of the naïve Bayes classifier, refer to Hand and Yu [15]. This approach was initially introduced for the diagnosis of bone tumors by Gwilym Lodwick [13]. Importantly, there is clear evidence that besides Lodwick growth grade, the parameters pretest probability, patient age, localization of the tumor, tumor size, and tumor matrix calcifications improve the predictive value [16–18].

### Artificial intelligence

In recent years, the advent of artificial intelligence (AI) applications in medical imaging has also impacted musculoskeletal oncological imaging [19]. AI decision support systems promise to reduce interobserver variability in reporting. Furthermore, they seem well-suited for evaluation of rare diseases like bone tumors, since extensive knowledge regarding bone tumor diagnosis cannot be taken for granted among general radiologists. Initial results of modern AI applications in the field of bone tumor diagnosis are promising [20, 21]. He and colleagues reported on a deep learning algorithm that is able to automatically classify bone tumors (benign versus malignant) as good as experienced readers [21]. It needs to be highlighted that the naïve Bayes classifiers used by Lodwick and colleagues [13], Kahn and colleagues [14], and Do and colleagues [5] are easy probabilistic machine learning (a branch of AI [22]) algorithms that process predefined, manually extracted features. In the near future, modern AI algorithms may potentially be employed as an automated second opinion reading. Cases in which the experienced musculoskeletal radiologist and AI algorithms disagree to an extent that patient management is dependent upon need to be discussed thoroughly. To date, the experienced musculoskeletal radiologist’s reading is still considered standard of care for interpretation of bone tumors on radiography.

### Radiography versus cross-sectional imaging

Estimation of growth rate of lytic bone tumors is performed to guide patient management [3]—i.e., decide whether a tumor can be regarded as benign, should be followed up, or whether biopsy is required. Although radiography is still considered the central diagnostic modality for bone tumors [17, 23], cross-sectional imaging modalities and additional functional imaging techniques have had tremendous impact on diagnostic evaluation and patient management in the last decades. MRI is superior to radiography regarding local staging of bone tumors [23]. FDG PET-CT successfully monitors treatment response in highly malignant bone tumors like osteosarcoma and Ewing’s sarcoma and may furthermore be utilized as a whole-body staging examination [24]. However, there is considerable overlap of quantitative FDG avidity of benign and malignant bone tumors. For the prediction of tumor type, FDG PET does therefore not replace morphological imaging but should be regarded as an adjunct [24].

## Conclusions

In this review, the original Lodwick algorithm for grading growth rate of lytic bone tumors on conventional radiography is summarized and visualized with an easy-to-use decision tree. Subtle details of the grading system that are easily being missed during everyday practice are highlighted to avoid misinterpretation. Introduction of Figs. 1 (decision tree) and 2 (exemplary images/ atlas) into clinical practice may reduce misapplication and increase reproducibility of growth rate grading. We hope the decision tree represents a valuable tool for easy and correct application of the Lodwick grading algorithm for students, for residents in radiology and for experienced musculoskeletal radiologists alike.

## Supplementary Information

Below is the link to the electronic supplementary material.

Supplementary file1 (DOCX 16 KB)
