# Supplementary material for: The Lodwick classification for grading growth rate of lytic bone tumors: a decision tree approach
Source: Skeletal Radiol. 2021 Jul 24;51(4):737–45. doi: 10.1007/s00256-021-03868-8 (PMC8854272; doi:10.1007/s00256-021-03868-8)
Supplement: Supplementary file 1 — Supplementary file1 (DOCX 16 KB) [file 256_2021_3868_MOESM1_ESM.docx]

Predefined expressions for grading growth rate of lytic bone tumors – supplemental material to “The Lodwick Classification for grading growth rate of lytic bone tumors: a decision tree approach”

In the following expressions, options are highlighted in orange.

A lytic bone tumor of *diameter1* x *diameter2* cm is observed in the *localization*.

Pattern of bone destruction: *geographic/moth-eaten/permeative (includes partly permeative)*

Margin: *regular/lobulated/multicentric/ragged/poorly defined/moth-eaten ≤ 1cm/moth-eaten > 1cm*

Penetration of cortex: *absent or partial/total*

Sclerotic rim: *present/absent*

Expanded shell: *absent or ≤ 1cm/>1cm*

**Grade IA**

- The tumor is geographic and has a *regular/lobulated/multicentric* margin. There is *no/partial* penetration of cortex, a sclerotic rim is observed. There is *no/a slightly* expanded shell. Therefore, Lodwick growth grade IA is assigned.

**Grade IB**

- The tumor is geographic and has a *regular/lobulated/multicentric* margin. There is *no/partial* penetration of cortex, a sclerotic rim is observed. There is an expanded shell > 1 cm. Therefore, Lodwick growth grade IB is assigned.
- The tumor is geographic and has a *regular/lobulated/multicentric* margin. There is *no/partial* penetration of cortex. There is *no/an incomplete* sclerotic rim observed. Therefore, Lodwick growth grade IB is assigned.
- The tumor is geographic and has a *ragged/poorly defined* margin. There is *no/partial* penetration of cortex. Therefore, Lodwick growth grade IB is assigned.

**Grade IC**

- The tumor is geographic and has a *regular/lobulated/multicentric/ragged/poorly defined* margin. There is total penetration of cortex. Therefore, Lodwick growth grade IC is assigned.
- The tumor is geographic and has a moth-eaten margin ≤ 1cm. Therefore, Lodwick growth grade IC is assigned.

**Grade II**:

- The tumor is geographic and has a moth-eaten margin > 1cm. Therefore, Lodwick growth grade II is assigned.
- The tumor shows a moth-eaten pattern of bone destruction. Therefore, Lodwick growth grade II is assigned.

**Grade III**:

- The tumor shows a permeative pattern of bone destruction / parts of the tumor show permeative bone destruction. Therefore, Lodwick growth grade III is assigned.
